# A randomized controlled study comparing the objective efficacy and safety of a novel self-inserted disposable vaginal prolapse device and existing ring pessaries

**DOI:** 10.3389/fmed.2023.1252612

**Published:** 2023-09-26

**Authors:** Elan Ziv, Tsvia Erlich

**Affiliations:** ConTIPI Medical Ltd., Caesarea, Israel

**Keywords:** pelvic organ prolapse, non-surgical management, disposable vaginal device, self-inserted device, ring pessaries

## Abstract

**Introduction:**

ProVate is a novel, disposable, collapsible self-inserted vaginal device for the nonsurgical management of pelvic organ prolapse (POP). We assessed possible vaginal microflora changes and POP reduction using ProVate and a commercially available ring pessary (control).

**Methods:**

We performed post-hoc analysis of data obtained from an interventional, prospective, multicenter, open-label, randomized, controlled, statistically powered (noninferiority), home-use, cross-over study conducted at seven sites. Safety and performance data collected for both devices were analyzed to compare objective POP reduction (employing the Pelvic Organ Prolapse Quantification System [POP-Q]), safety (assessed by the incidence of adverse events [AEs]), and the rates of certain AEs.

**Results:**

Eighty-five women with symptomatic POP were screened; 71 were randomized, and 58 completed the study per protocol. Forty-nine (90.7%) ProVate users experienced complete prolapse reduction (stage 0), 3 (5.6%) experienced reductions to POP-Q stage 1, and 2 (3.7%%) experienced reductions to stage 2. Collectively, 52/54 (96.3%) ProVate users experienced prolapse reduction to stage 0 or 1. In all, 47/57 (82.5%) control users experienced complete prolapse reduction, while 5 (8.8%), 4 (7.0%), and 1 (1.8%) experienced reductions to stage 1, 2, and stage 3, respectively. Collectively, 52/57 (91.2%) control users experienced reductions to either stage 0 or 1. In 53/54 (98.1%) ProVate and 55/57 (96.5%) control users, there was at least 1 POP-Q stage prolapse reduction, and in 32 (91.4%) ProVate and 31 (83.8%) control users who had stage ≥3 prolapse, there were at least three POP-Q stage reductions. In total, 26/71 (36.6%) ProVate and 22/64 (34.4%) control users in the safety population experienced AEs. The incidence of device-related AEs was 17/71 (23.9%) for ProVate and 13/64 (20.3%) for the control. Most AEs were minor, mild, and anticipated.

**Conclusion:**

Our analysis demonstrated that ProVate and the control are highly effective in reducing POP, and both are associated with comparably low numbers of AEs. However, ProVate has the advantage of being more user-friendly, suitable for home use, and expected to allow women with POP to practice better and easier self-care.

## 1. Introduction

Pelvic organ prolapse (POP) of some degree affects up to 75% of US women who delivered vaginally, depending on the method of reporting (symptoms, pelvic exams, or both) (1, 2). Only 3–8.3% of these patients are symptomatic and consult with experts regarding available treatments (3–6). Of them, 210,000–300,000 women with the condition are operated on annually, while the rest either use pessaries or remain untreated.

Currently available ring pessaries are the most widely used type of pessary, considered effective, and safe, but also associated with substantial downsides (7–9) which limit their widespread use; they are all reusable only, hard, resilient large bodies. Insertion and removal are done manually, in their large dimensions, often being difficult, painful, or unpleasant (10), most often necessitating a medical practitioner, with dependency upon clinic visits (usually every 3 months). They are associated with many adverse events (AEs) such as irritation, discharge, infections, vaginal wall trauma, etc. (11). There is also a high rate of discontinuation, which exceeds 50% (range: 37–80%) (12–15) within 12 months, with the main reasons being the inability of users to insert and remove the device, failure to retain (16, 17), discomfort (18), pain during insertion or removal (19), desire for another treatment modality (e.g., surgery) (20), dependency on clinic visits (21), and sexual disturbances (22, 23). However, we note that several users do not experience any AEs, are satisfied with this management, and continue usage for years.

The huge gap between existing cumbersome pessary management, and women’s wish for a more pleasant and comfortable home-use POP control and for unhindered intercourse, dictated the development of a new device, ProVate, which retains the upsides of a ring pessary while substantially reducing or eliminating its major downsides. The device was designed to be used by lay women, while the applicator leads the device into the correct position within the vagina, thus eliminating the need for constant replacement appointments. Notably, some women, primarily the elderly frail, will not be able to insert a ProVate device by themselves and will continue using the existing ring pessaries.

**Figure 1:**
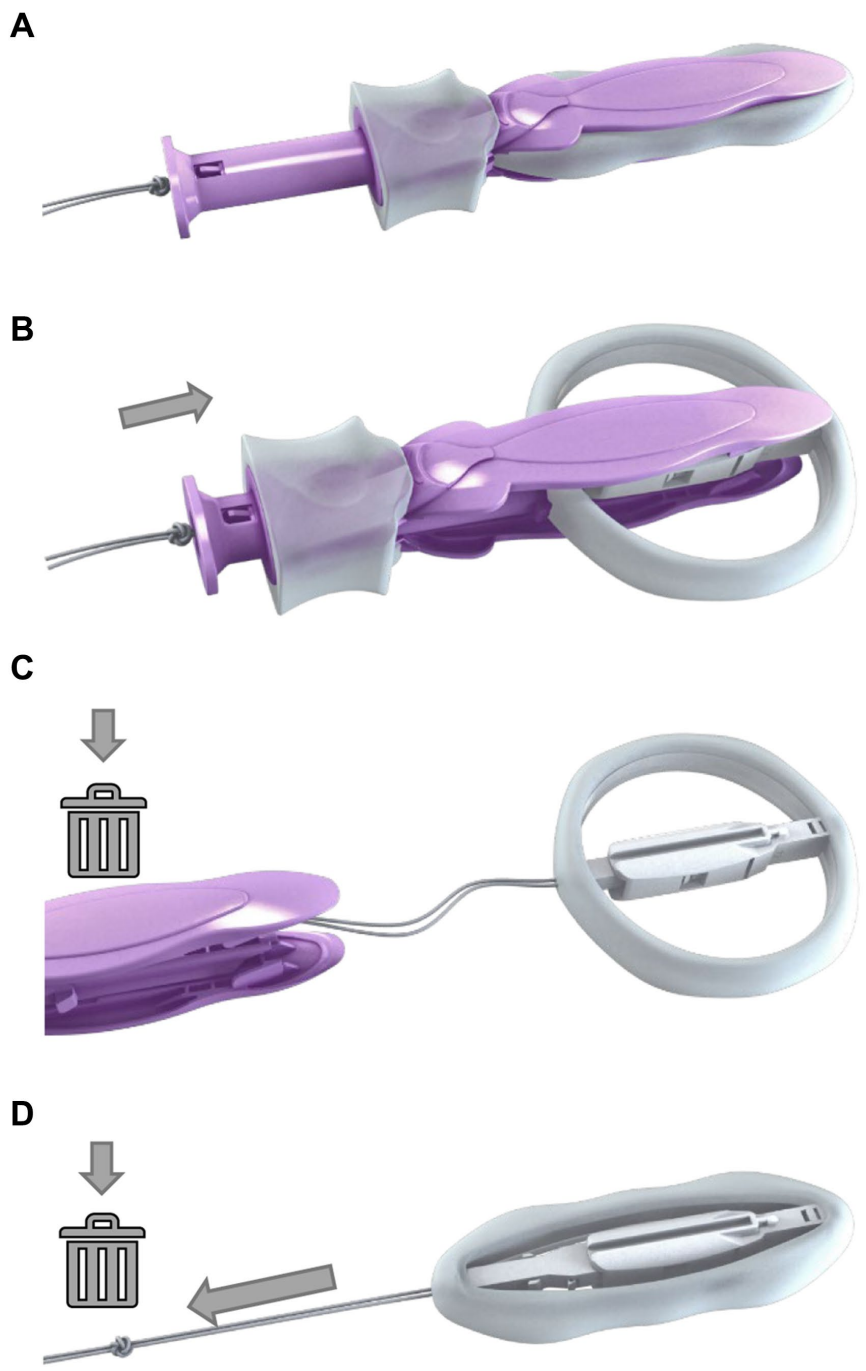
The ProVate Device with its various configurations during insertion and removal The ProVate device is provided clean, within a personal wrap, readily available for immediate vaginal insertion, in small dimensions, within a disposable applicator **(A)**. Following vaginal insertion, similar to insertion of the menstrual tampon, the plunger is pushed and the slender compacted device within the applicator gradually enlarges to become a ring **(B)**. By the end of pushing of the plunger, the ring becomes fully deployed and the applicator separates from the ring, and is removed from the vagina for disposal, leaving the string available for later removal **(C)**. The deployed ring may remain in the vagina for up to 7 days, when a pull on the string collapses the ring into its slender pre-insertion size, for comfortable removal and disposal **(D)**. (With permission from ConTIPI Medical Ltd.).

ProVate (Figure 1) was designed to perform like the ring pessary by lifting up various vaginal walls when in place while being suitable for home use. This is a disposable ring pessary (24), provided ready for use (with six different sizes) within an applicator, in small dimensions, and a slender shape, which becomes a full-size ring pessary when already within the vagina. ProVate may remain within the vagina for up to seven days, when a pull on the string minimizes the ring’s diameter to regain its pre-insertion slender shape, and the device is removed for disposal. The user may insert another device immediately or later as she so wishes.

The objective of the study was to confirm that ProVate does not alter the vaginal microflora in a clinically significant manner compared to the commercially available ring pessary (control). We also evaluated the effectiveness and safety of ProVate and the control.

## 2. Methods

### 2.1. Study design

The study was designed as an interventional, prospective, multicenter, open-label, randomized, controlled, statistically powered (non-inferiority), home-use trial, testing ProVate, and the control in a sequential cross-over fashion. The study was conducted in seven outpatient gynecology/urogynecology clinics (six in the US and one in Israel) over 14 months between August 2017 and September 2018.

The study received ethical approval from the Institutional Review Board Service in the US (#Pro 00022375), and from Assuta Medical Ethics Committee in Israel (#2016028), and all participants gave their written informed consent.

According to the study’s usage period, each participant received either a clean sealed disposable ProVate (ConTIPI Medical, Caesarea, Israel), or a new reusable ring pessary (not using their used device) made by a single US manufacturer (Ring with support by Milex®, Cooper Surgical Inc., Trumbull, CT).

**Figure 2:**
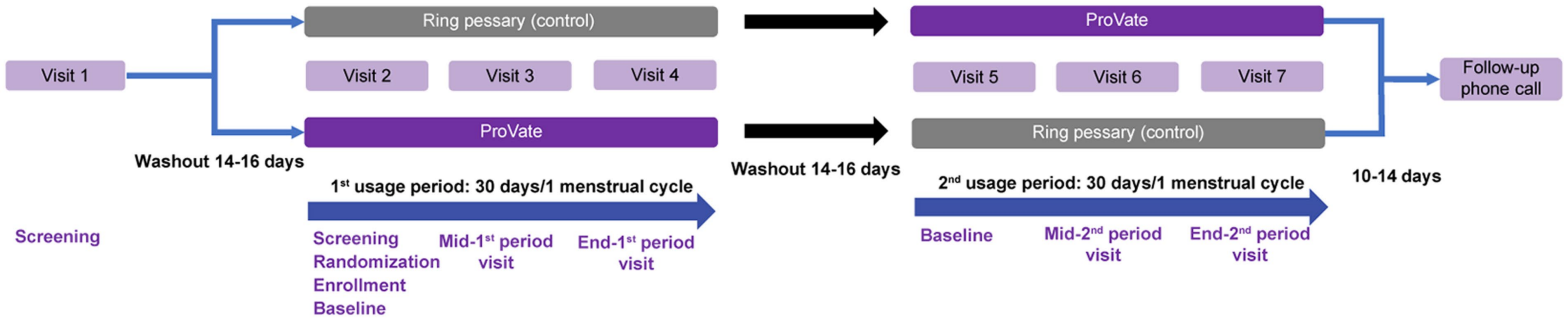
Study design. Screening started at visit 1, followed by a 14–16-day washout period. Visit 2 was a baseline visit for the first usage period, with screening, randomization into either the ProVate group or the control group, and enrollment of eligible participants. Visit 3 was a mid-period visit to assess compliance with study restrictions, and visit 4 was the end-visit of the first usage period, and results from this visit were compared with those from visit 2. Following another 14–16-day washout period, the second usage period began, during which each subject used the alternative studied device (visits 5–7) for the same length of time. A follow-up phone call to subjects ended the study 10–14 days after visit 7. (With permission from ConTIPI Medical Ltd.).

After the screening phase, the first usage phase (Figure 2) started following a 14–16-day washout period in which participants were requested to refrain from using any vaginal device and comply with study restrictions till the end of the trial. During the second visit, screening was completed, participants were randomized in a 1:1 ratio into either group A (using ProVate first and then the control) or B (using the control first and then ProVate), and size-fitting for each group was done. The device’s usage period was 30 ± 3 days for post-menopausal participants or the length of each participant’s menstrual cycle ±3 days (range: 26–40 days) for menstruating participants, and ended at visit #4, while visit #3 was a confirmatory visit to ascertain sizing and adherence with the study protocol. After visit #4, there was another 14–16-day washout period for post-menopausal participants or one menstrual cycle for pre-menopausal participants. The second usage period began with the same follow-up but with the alternate device following another round of sizing. This was followed by the regular use of the chosen size for 30 ± 3 days for post-menopausal participants or the length of a given participant’s menstrual cycle ±3 days (range: 26–40 days) for menstruating participants, and ended at visit #7, while visit #6 was a confirmatory visit.

If the device was either too small (expulsion) or too large (discomfort), it was refitted. During the ProVate usage period, participants were instructed to use as many devices as they wished for 1–7 days and fill in a daily diary, documenting each device’s usage length, functionality, and AEs. The ProVate usage period was at least 18 consequent or non-consequent usage days in the PP set, while the control ring pessary remained *in situ* during the entire study period. During each clinic visit, participants were examined vaginally to assess the stage of prolapse according to the POP-Q scale, with or without the device, and to check for the presence of AEs.

### 2.2. Inclusion/exclusion criteria & study restrictions

We included females aged 21–80 years who were diagnosed with POP-Q stage 2–4 vaginal prolapse in one or more sites along their vaginal walls, women who previously used a vaginal ring pessary, women who could use both hands and insert a device into their vaginas, and women in whom a 61-91-mm pessary (or an equivalent-sized control) could be well-fitted and retained.

We excluded patients with previous inability to accommodate tampons or vaginal pessaries, women who were currently participating in another clinical study, patients with a comorbid condition(s) or severe systemic diseases that could limit their ability to participate in the study, pregnant women, women with suspected pregnancies, or the intention to get pregnant during the study period, women with abnormal vaginal bleeding in the previous six months, women who underwent vaginal surgery during the preceding three months, women with severely atrophic vaginas, existing vaginal or vulvar lacerations, and symptomatic vaginal, or urinary tract infections as determined by physical examinations and lab results, women who used medications (corticosteroids, antibiotics, etc.) and had medical conditions that may have compromised their immune systems, and women with recurrent urinary tract infections and abnormal cervical cytology.

All participants were instructed to avoid activities or the use of commercial products that may modify the vaginal flora, such as vulvar., or intimate cosmetics, medications, contraceptives, and wipes. They were also to avoid any vaginal devices other than the study devices and use only the menstrual supplies and condoms provided by the study site.

### 2.3. Pop reduction & safety outcomes

Data collection at baseline (no device) and at the final visit included the Pelvic Organ Prolapse Quantification (POP-Q) staging for each subject and AE recording. These data were further analyzed post-hoc with two objectives: (I) to show that the objective reduction of POP while using ProVate is high and comparable to the control; and (II) to show that safety, as assessed by the rate of AEs for both ProVate and control, is comparable and that the rates of certain AEs are expected to remain low once insertion and removal are in small dimensions and a disposable device with a limited usage period is used.

Efficacy was assessed using two performance indicators:

The proportion of participants who had their prolapse reduced to POP-Q stage 0/1/2/3 while using ProVate or the control, regardless of the initial POP-Q stage (the first objective efficacy outcome).

The proportion of participants who had 1, 2, or 3 POP-Q stage reductions while using ProVate or the control, compared to baseline, using the PP analysis set (the second objective efficacy outcome).

POP-Q staging was determined at baseline as well as at the last visit with each of the studied devices. The percentage of participants who had their prolapse reduced to POP-Q stage 0/1/2/3 while using ProVate or the control, regardless of the initial POP-Q stage, while using the PP analysis set, was determined. The percentages of participants who had, 1, 2, or 3 POP-Q stage reductions while using ProVate or the control, were also evaluated.

Safety was assessed by recording the incidence of anticipated and unanticipated AEs. Anticipated AEs included vaginal wall trauma, vaginal/urine infections, pain, spotting, discomfort, *de novo* urinary incontinence, and constipation.

AEs were obtained and reported using all of the following methods: daily diaries, direct questioning, and vaginal examination during all routine study visits, scheduled weekly telephone calls to participants, and non-scheduled calls from participants.

### 2.4. Statistical methods

The safety analysis set (SA) was defined as all randomized subjects who used at least one device (ProVate or the control) for any duration [Full Analysis set (FA)].

The Per-Protocol analysis set (PP) included all randomized participants who (I) used ProVate and the control for at least 16 days out of each device usage portion of the study, (II) had no major protocol deviation, and (III) had no evidence of vaginal infection at enrollment. Safety analyses were conducted on the safety/FA population and efficacy analyses were conducted on the PP population.

Statistical analyses were performed using SAS v 9.4 (SAS®, SAS Institute Cary, NC USA) software. All statistical tests and confidence intervals were two-sided and tests were performed using a 5% significance level.

Categorical analyses of the percentage of POP-Q responders were performed using Fisher’s exact test. The post-hoc analyses presented here show analyses of differences between the groups with *p*-values and 95% confidence intervals (CI) of rate differences. The percentage rate differences and 95% CI of the differences based on the Farrington-Manning Score tests are presented in the results section.

## 3. Results

**Figure 3:**
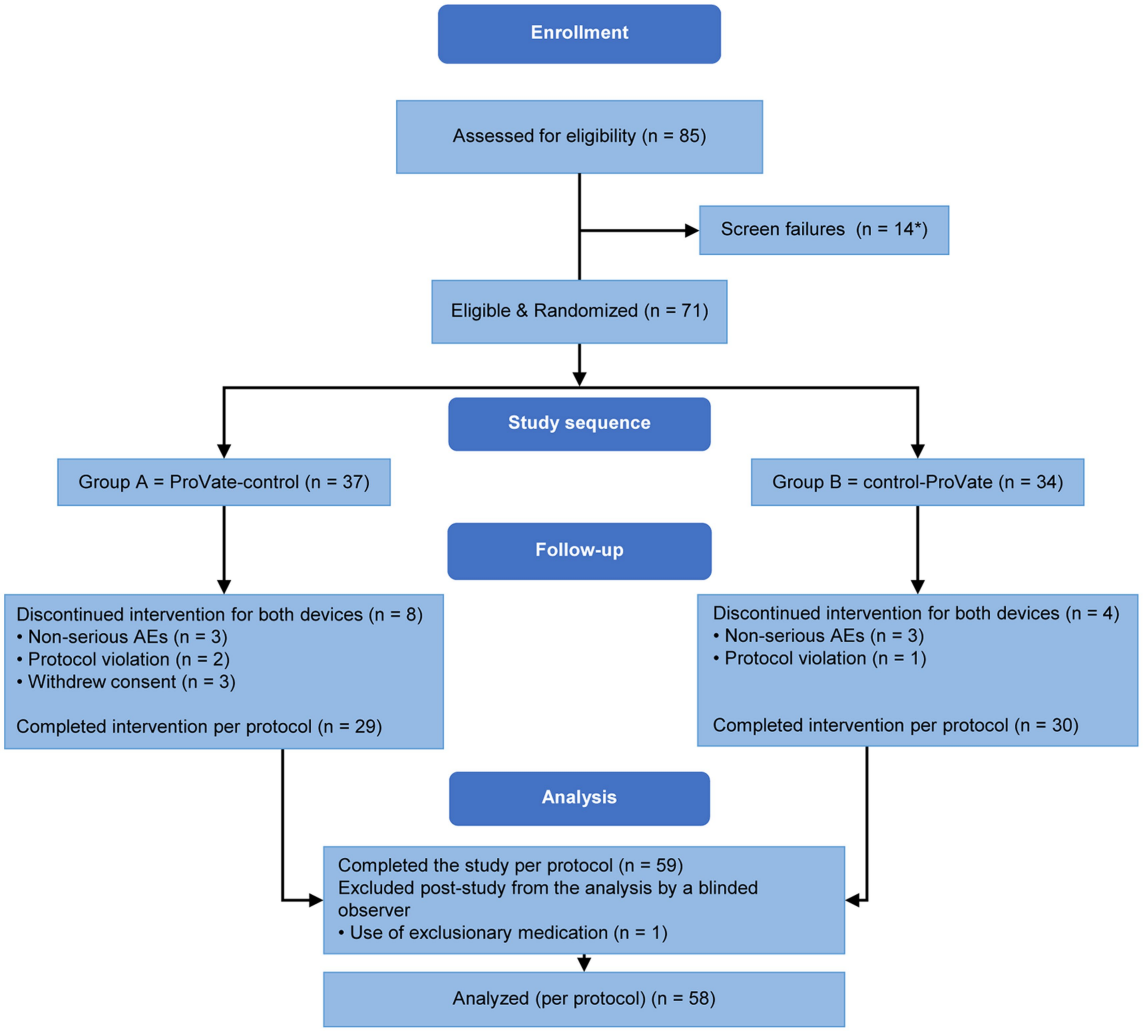
Subject disposition. Eighty-five women enrolled and screened. Seventy-three of them were randomized into either group A (ProVate-control) or group B (control-ProVate). Fifty-nine participants completed the study per protocol; however, one’s data were excluded by a blinded reviewer because she used an exclusionary medication. Hence, the data of 58 participants were analyzed per protocol. [with permission from ConTIPI Medical Ltd.]. *Two participants were randomized but did not meet our inclusion criteria requiring the ability to use one of the available sizes in the study (ProVate or Control); hence, we considered them screening failures.

Symptomatic participants accustomed to using ring pessaries were recruited from seven community gynecology/urogynecology clinics. Eighty-five women with symptomatic POP were screened (Figure 3); 73 were randomized and 58 completed the study per protocol. At screening and before the introduction of any vaginal device, 21 (36.2%) of the 58 subjects in the PP population had POP-Q stage 2 prolapse, 35 (60.3%) had POP-Q stage 3 prolapse, and 2 (3.4%) had POP-Q stage 4 prolapse.

The general characteristics of our study participants are summarized in Table 1.

**Table 1:** Characteristics of our study participants.

		Participant’s characteristics
Mean age (Years)		64.5 ± 10.5
Body mass index		28.2 ± 5.5
Menopausal status (*N*, %)	Postmenopausal	53 (91.4%)
	Premenopausal	4 (6.9%)
	Perimenopausal	1 (1.7%)
Systemic HRT use (*N*, %)		4 (6.9%)
Vaginal estrogen use (*N*, %)		5 (8.6%)
Prestudy POPQ-Q staging	2	21 (36.2%)
	3	35 (60.3%)
	4	2 (3.4%)

Participants were allowed to use as many ProVate devices as they wanted for at least 24 h and up to seven days; however, they were encouraged to use ProVate for as long as possible within those limits. In total, 383 ProVate devices were used in the safety population, a mean value of 5.7 ± 1.6 devices per user, and 350 ProVate devices in the PP population, a mean value of 6.0 ± 1.1 devices per user. In 62.8% of the participants, a single ProVate device was used for at least four days, whereas a single reusable control remained in the vagina during the entire control phase (≥23 days) in 89.6% of the participants. The total number of usage days was 1,647 for ProVate and 1,734 days for the control (PP population).

No significant effect of sequence randomization was observed (*p* = 0.325), suggesting the lack of a sequence effect and allowing the pooling of the results for each device from both sequences in all analyses.

### 3.1. Objective efficacy – the reduction of the POP stage (PP population)

Objective efficacy analyses were conducted on the PP set with a total of 58 participants. In the ProVate group, four cases of end-study POP-Q results were missing; hence, POP reduction was calculated over 54 cases. In the control group, one case of end-study POP-Q results was missing; hence, POP reduction was calculated over 57 cases.

Both ProVate and the control, once deployed, function as a mechanical intravaginal scaffold with an immediate lift-up of the vaginal apex and distension of its lateral walls. Our study population included participants with multiple-site prolapse (e.g., anterior, and apical). However, POP reduction while using ProVate or the control was not limited to a specific site (e.g., apical). Furthermore, the upward distension of the vaginal apex caused flattening of the vaginal walls as well as prolapse reduction at other sites.

The objective efficacy outcomes for both ProVate & the control are further presented in Table 2.

**Table 2:** Prolapse reduction while using ProVate (54 subjects) and the control (57 subjects) in the per-protocol population.

POP reduction results with ProVate or control (by POP-Q staging, *N* = 58)		Study results	
		ProVate *N* = 54^*^	Control *N* = 57^**^
First Objective Efficacy Outcome Final POP-Q Stage Achieved with ProVate/Control at End Visits (% Subjects)	POP-Q stage 0 while using ProVate or control	90.74%	82.46%
	POP-Q stage 1 while using ProVate or control	5.56%	8.77%
	POP-Q stage 2 while using ProVate or control	3.70%	7.02%
	POP-Q stage 3 while using ProVate or control	0%	1.75%
Second Objective Efficacy Outcome Prolapse Reduction (# of Stages Reduced)	% of subjects with ≥1 stage POP reduction from baseline	98.15%	96.49%
	% of subjects with ≥2 stages POP reduction from baseline	94.44%	87.72%
	% of subjects with ≥3 stages POP reduction from baseline	91.43%	83.78%

* Four results were missing in the PP population.

**One result was missing in the PP population.

**Figure 4:**
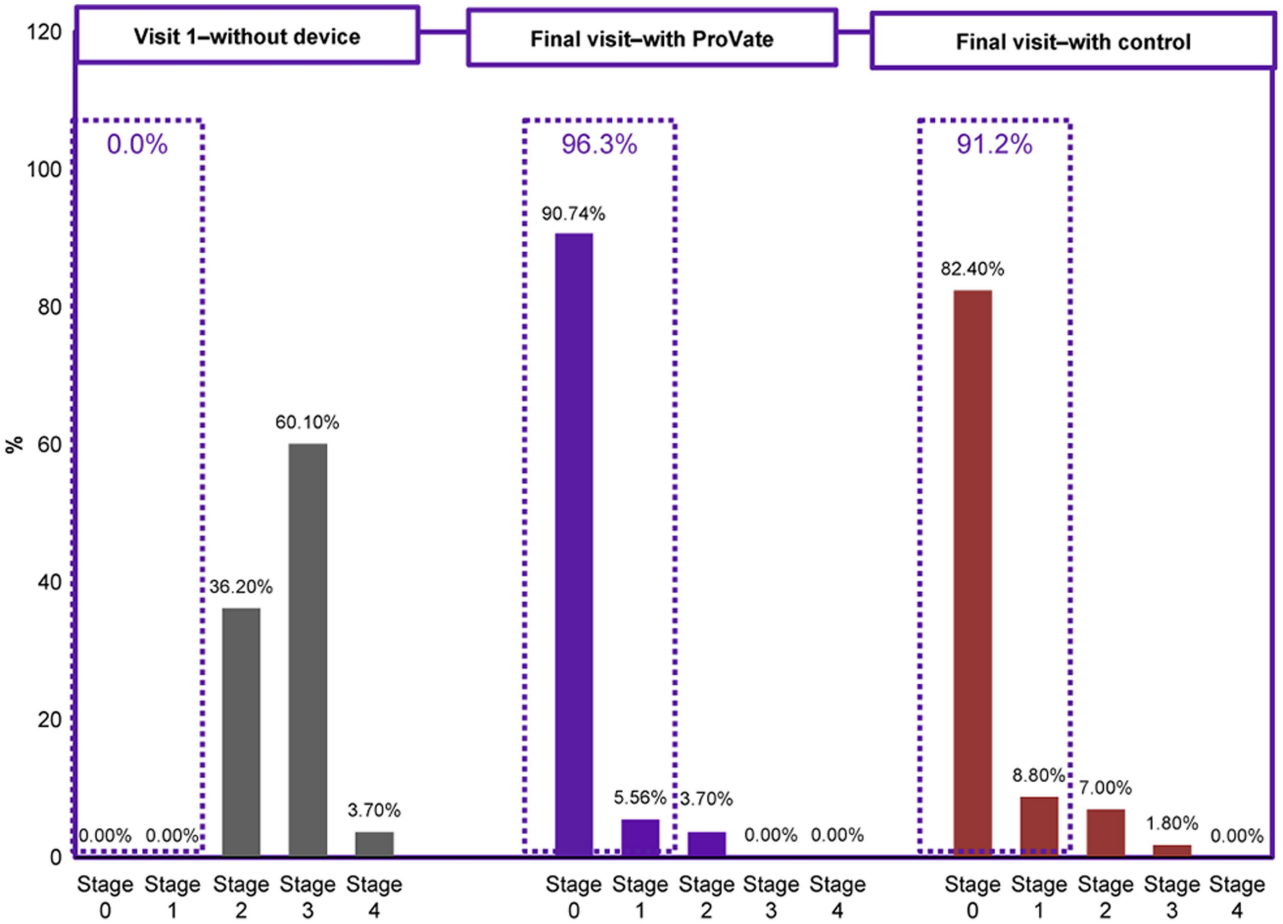
Comparison of the objective efficacy (POP-Q staging) before using any vaginal device and while using either ProVate or the control. Before the onset of the study, all participants had POPO-Q stage 2–4 prolapse. While using ProVate, 96.3% of participants had a substantial reduction of prolapse to POP-Q stage 0/1. While using the control (a market-available ring pessary), 91.2% of participants had a substantial reduction of prolapse to POP-Q stage 0/1. There was no statistically significant difference detected between results in the ProVate group and those in the control group (*p* = 0.4391).

The first objective efficacy outcome relates to the proportion of participants who had either POP-Q stage 0, 1, or 2 prolapses at the final visit, while using the ProVate, or the control, using the PP analysis set. While using ProVate, 49 participants (90.7%) experienced complete prolapse reduction (to stage 0), 3 participants (5.6%) experienced prolapse reduction to POP-Q stage 1, and 2 participants (3.7%) experienced prolapse reduction to stage 2 (Figure 4). Collectively, 52 of the 54 ProVate cases (96.3%) experienced prolapse reduction to either stage 0 or 1 which is usually no longer symptomatic. With the control, 47 participants (82.5%) experienced complete prolapse reduction (to stage 0), 5 participants (8.8%) experienced prolapse reduction to POP-Q stage 1, 4 participants (7.0%) experienced prolapse reduction to stage 2, and one participant (1.8%) experienced prolapse reduction to stage 3. Collectively, 52 of the 57 cases (91.2%), experienced prolapse reduction to either stage 0 or stage 1. We detected no statistically significant difference between the proportion of non-symptomatic participants in the ProVate group relative to those in the control group (96.3% vs. 91.2%, *p* = 0.4391). The difference between the ProVate and control groups was 5.1[95% CI: −3.8, 14.0]%.

The second objective efficacy outcome relates to the proportion of participants who had 1, 2, or 3 POP-Q stage reductions while using ProVate or the control. Following the insertion, in 53/54 (98.1%) of ProVate users and 55/57 (96.5%) of control users, there was at least a one-POP-Q-stage prolapse reduction. The difference between the ProVate and control groups was 1.7[95% CI: −4.3, 7.6]% (value of *p* = 1.00). In the ProVate and control groups, there were at least 2 POP-Q stages of prolapse reduction in 94.4 and 87.7% cases, respectively. The difference between the ProVate and control groups was 6.7 [95% CI: −3.8, 17.2]% (value of *p* = 0.3225). Of the 35 ProVate and 37 control participants who had POP-Q stage 3 or 4 during the screening, 32 ProVate (91.4%) and 31 control (83.8%) participants had at least 3 POP-Q stages of prolapse reduction. The difference between the groups was 7.6[95% CI: −7.4, 22.7]% (value of *p* = 0.4799). No statistically significant differences (all *p*-values >0.05) were found between the ProVate and control groups for 1, 2, or 3 POP-Q stage reductions.

### 3.2. AEs

General safety analyses were conducted on the SA set with a total of 71 participants with ProVate and 64 with the control. There were 26 out of 71 (36.6%) ProVate participants and 22 out of 64 (34.4%) control participants in the safety population who experienced any AE. A summary of the categorization of AEs is shown in Table 3. Per this summary, the percentage of total AEs did not differ significantly between the two devices.

**Table 3:** Comparison of the incidence of AEs between ProVate and the control.

Adverse events	ProVate *N* = 71	Control *N* = 64
Total AEs/participants (% of FA)	54/26 (36.6%)	31/22 (34.3%)
Users with device related AEs	17/71 (23.9%)	13/64 (20.3%)
Anticipated device related AEs (PP set)	32/40 (80%)	9/17 (52.9%)
Serious device-related AEs	None	None
Resolution without sequelae	54/54 (100%)	26/31 (83.8%)
Number of devices used	383/73 participants	1 per each participant
Total length of use	1,647 days	1734 days

Table 4 shows the distribution of all 57 device-related AEs which occurred while using both devices – 383 ProVate devices used over 1,647 usage days, and a single ring pessary for each woman, altogether tried for 1734 usage days. The largest part of the AEs list, for both devices, consists of sporadic AEs, usually of 1–2 complaints each.

**Table 4:** Distribution of device-related AEs while using ProVate and the control (AEs/users with AEs).

Body system	Complaint	ProVate		Control	
		Anticipated	Non-anticipated	Anticipated	Non-anticipated
Abdomen	Pain	1	3/2		1
	Tenderness		1		
Back	Pain		1		
Urinary Tract	Urgency		1		
	Frequency	1			
	De-Novo SUI			1	
	UTI			1	1
Pelvic	Discomfort	1	1		1
	Pain		1		
Vaginal	Discharge	3/2		3/3	2/2
	Wall trauma	5/4		2/2	
	Spotting	8/6		1	
	Odor	2/2			
	Infection			1	
	Granulation tissue	1			
	Discomfort	8/6			
	Vulvovaginal pain	1			
	Swelling	1			
	Itching				2/2
Vulvar	Itching				1
Number of AEs/number of users		32/16	8/4	9/7	8/6
Total number of AEs/number of users		40/17		17/13	

The most common AEs with ProVate were vaginal discomfort and vaginal spotting (eight cases in six participants for each complaint during the study), which are anticipated for all devices that are used vaginally. The most common AE with the control was vaginal discharge (five cases in five participants), which is also anticipated for many devices that are used vaginally for long periods.

With ProVate, there were no signs nor symptoms of vaginal or urinary tract infections (UTIs), by both self-report, and vaginal examinations, during the study. With the control, there was one case of vaginal infection, two cases of bothersome vaginal complaints, and one case of UTI requiring treatment with antibiotics.

There were five cases of vaginal wall trauma in four women using ProVate and two cases in two women using the control, again, a nonsignificant difference.

## 4. Discussion

The aim of this post-hoc analysis of data from a randomized controlled trial was to demonstrate the substantial objective efficacy and safety of ProVate in the non-surgical management of POP and compare it to the efficacy and safety of a commercially available ring pessary (the control). ProVate and the control reduce POP in the same manner (provide mechanical support); yet, ProVate has the following upsides: it is an all-disposable device, can be self-inserted and removed by the user, can be used anywhere, and at any time, has small dimensions, exists within an applicator, and comes with no dependency on the clinic (other than routine checkups); thus, it is expected to improve user’s experience and substantially reduce the discontinuation rate (25) while enabling unhindered pessary usage even during times of inability to attend the clinic for ongoing pessary replacement [e.g., frailness, exacerbations of severe illnesses, pandemics (26, 27), etc.], which may increase the incidence of AEs.

There is no common definition for “success” in POP reduction either with surgery or with non-surgical methods. Barber et al. (28) defined the rate of surgical success when all anatomic supports were proximal to the hymen to have the lowest treatment success rate (19.2–57.6%), while there was a 94% surgical success rate when the definition was the absence of prolapse beyond the hymen. Miceli and Duenas-Diez (29) found the success rates of pessaries and surgery in prolapse reduction to be comparable (84.4 and 98.6%, respectively) where success was defined as no prolapse >POP-Q stage 2.

This definition of treatment success, when a woman still has a POP-Q-stage-2 prolapse, may be too lenient; thus, attempts should be made to reduce the prolapse to POP-Q stage 1 or even 0, which, in most cases, is asymptomatic. Therefore, we propose that objective treatment success reports should follow Table 2 in this analysis, reporting the percentage of users with their post-treatment POP-Q stages achieved and the percentage of users with certain POP-Q stages reduced.

ProVate is a disposable flexible vaginal ring pessary designed to overcome many downsides of existing ring pessaries. This study demonstrates that ProVate, once deployed, reduces POP as much as the control. Both devices (ProVate and the control) function as a mechanical intravaginal scaffold with an immediate lift-up of the vaginal apex and distension of its lateral walls; therefore, it also reduces any prolapse at other vaginal sites (e.g., stage 3 anterior wall prolapse, stage 2 uterine prolapse, and stage 1 posterior wall prolapse, in the same woman, may end as a POP-Q stage 0 while using a properly fitted ProVate or the control). In other words, both devices are expected to provide similar support and uplift to all vaginal walls; however, ProVate also provides its unique qualities, allowing women to manage their POP with a home self-use disposable device that has a tampon-like applicator for self-insertion.

A search of the literature revealed only scarce previous evidence on objective ring pessary efficacy, as demonstrated by the proportion of POP-Q stage reduction(s) (e.g., 1/2/3 stage reductions from the baseline), and by eventual POP-Q stage achievement with a pessary (e.g., reduction to stage 0/1/2). Many reports use parameters such as the discontinuation rate, quality of life, and subjective efficacy questionnaires as indicators of efficacy (30). In a previous ProVate study (24), 97.83% of ProVate users had POP-Q stage 0 (no prolapse) and 2.17% of them had POP-Q stage 1 prolapse by the end of the study (100% substantial reduction).

The results of the current study add new evidence as to the objective efficacy of ring pessaries. With ProVate, in 96.3% of participants, there was prolapse reduction to POP-Q stage 0 or 1, over 98% of them had a one-stage prolapse reduction, and in over 91% of them, there was even a three-stage reduction. Similar results were found with the control, whereby in 91.2% of patients, there was prolapse reduction to POP-Q stage 0 or 1, over 96% of patients had a one-stage prolapse reduction, and in over 83% of them, there was even a three-stage reduction.

In the current study, the rate of total AEs/participants with complaints did not differ significantly – 54 AEs in 26 participants (26/71 = 36.6%) with ProVate and 31 AEs in 22 participants (22/64 = 34.3%) with the control (Fisher’s exact *p*-value = 0.8578). The rate of device-related AEs for both devices was rather low when compared to the literature [citing up to 73.1% of AEs (31)], with 40 AEs in 17/71 (23.9%) participants for ProVate and 17 AEs in 13/64 (20.3%) participants for the control. Most AEs were minor, mild, and anticipated.

In a previous study on ProVate (24), There were 91 AEs in 51/111 participants (45.95% of the FA set), probably due to the unique AE reporting system with a daily diary and direct questioning every week, which is likely to lead to a much larger proportion of complaints. In that study, 98.9% of AEs were mild and 87.9% of them were anticipated. Up to 58.9% occurred during the first week, and 75.8% occurred during the use of the first five devices.

When a new vaginal device is introduced, a learning/accommodation period, during which participants become accustomed to the device, is expected. In a previous ProVate study (24), it was also reported that most anticipated AEs with ProVate occurred during the sizing phase and during the beginning of the usage phase and reduced while participants became more experienced with device self-usage.

Also, the initial usage of any intravaginal device, including menstrual tampons, and mainly with estrogen deprivation, may be accompanied by some discomfort and spotting. As anticipated, these two were the most prevalent AEs with the initial usage of ProVate, though still of minimal low frequency, further diminishing with use.

Two AEs that may cause some concern with pessary use are further discussed below:

Vaginal wall trauma, a well-known, and described AE of pessaries, occurs in up to 24% of pessary users (32). In the current study, it occurred in 4/71 (5. 6%) ProVate users and in 2/64 (2.8%) control users. The difference in the incidence of this AE between the two devices could be attributed to the fact that most women in the study were accustomed to the use of the control, which was inserted by a physician, for years, while it was their first experience with ProVate, with which there was a short learning curve effect; thus, more AEs were expected (e.g., vaginal wall trauma) during the initial attempts at its insertion. This mechanism of wall trauma is probably different from situations where trauma is caused by prolonged pressure (“pressure ulcers”) exerted by a pessary that remains in the vagina for lengthy periods.

Urogenital infections are common in women. Vaginal purulent discharge, pruritus, and foul smells are common among pessary users (33, 34). In this study, with ProVate, there were no signs, or symptoms of vaginal or UTIs. With the control, there was one case of vaginal infection, two cases of bothersome vaginal complaints, and one case of a UTI requiring antibiotics. Though small, the difference is worth noting.

The main strength of this study is that it is a randomized controlled study that compares two POP devices and shows that efficacy and safety results obtained with the ProVate are favorable and comparable with results from the same women when using the control device, which has been on the market for several decades. Other strengths of this report include the daily collection of data on patients’ experiences with the device and AEs in a daily diary over 1647/1734 usage days (compared to most other studies that rely on memory recall only), and the design of the study where strict patient follow-up was employed, with frequent interactions between patients and the research team, allowing for the early, strict and accurate detection of specific AEs (e.g., vaginal wall trauma) and prompt implementation of corrective measures (e.g., instructing participants on proper insertion of the device).

The main limitation of this report is the rather short follow-up period of as little as 33 usage days, which is certainly sufficient to demonstrate efficacy and safety but may not reflect the long-term usage habits of participants. This study was designed only to demonstrate that the efficacy and safety of ProVate are comparable to those of the control; however, it did not attempt to look into the specific advantages of each device over the other.

This post-hoc analysis demonstrates that ProVate and the control are both effective in reducing POP and are both associated with a (comparable) low number of AEs. However, ProVate has the advantage of easy self-handling (insertion and removal) and allows women to resolve their POP issues by themselves.

This analysis also raises the question as to the correct way of reporting the success of POP treatments, where the current definition of objective POP reduction may not be relevant anymore. The objective success rate should be defined as the number of POP-Q stages reduced, and the final POP-Q stage achieved.

## 5. Conclusion

ProVate is expected to provide a useful & beneficial solution to women, enabling them to self-manage their POP issues and have autonomy over their intimate behavior (e.g., allowing for device-free intercourse), with a single-use small-size device at insertion & removal, and without the frequent dependency on the clinic. Many healthcare organizations intend to manage certain medical conditions at home. In an era where clinic visits are avoided by many women (due to reasons such as pandemics), and where treatment avoidance may lead to complications, ProVate is an example of a simple and available self-use home management modality. Moreover, this new management modality for POP may increase POP treatment compliance among untreated patients with POP. Further studies will be required to learn more about other characteristics of this device.

## Data availability statement

The data analyzed in this study is subject to the following licenses/restrictions: The data that support the findings of this study are confidential assets of ConTIPI Medical Ltd. Restrictions apply to the availability of these data as they are the company’s confidential and restricted data; therefore, they are not publicly available. However, data may be available from the corresponding author upon reasonable request and with the permission of ConTIPI Medical Ltd. Requests to access these datasets should be directed to eziv@contipi.com.

## Ethics statement

The studies involving humans were approved by the Institutional Review Board Service in the US (#Pro 00022375), and Assuta Medical Ethics Committee in Israel (#2016028). The studies were conducted in accordance with the local legislation and institutional requirements. The participants provided their written informed consent to participate in this study.

## Author contributions

EZ: conceptualization, methodology, data collection as the PI, data analysis, and writing – original draft. TE: conceptualization, methodology, data analysis, and writing – original draft. All authors contributed to the article and approved the submitted version.

## Funding

ConTIPI Medical Ltd. (Caesarea, Israel) funded this study as part of its R&D/regulatory process. EZ and TE designed the study and contributed as employees and members of the research team. However, the study was conducted by an external CRO, and the statistical analysis was done by an external firm. Therefore, the funder had no involvement in these activities. ConTIPI Medical Ltd. also funded all costs associated with the writing and publication of this manuscript.

## Conflict of interest

EZ has a conflict of interest as he is an employee and shareholder of ConTIPI Medical, the company that developed the studied device. EZ took part in the design of the study, data collection, interpretation of the results, and manuscript writing. TE has a conflict of interest as she is a consultant and optionee at ConTIPI Medical, the company that developed the studied device. TE took part in the design of the study, interpretation of results, and manuscript writing.

## Publisher’s note

All claims expressed in this article are solely those of the authors and do not necessarily represent those of their affiliated organizations, or those of the publisher, the editors and the reviewers. Any product that may be evaluated in this article, or claim that may be made by its manufacturer, is not guaranteed or endorsed by the publisher.
